# Advanced Magnetic Resonance Imaging (MRI) Techniques: Technical Principles and Applications in Nanomedicine

**DOI:** 10.3390/cancers14071626

**Published:** 2022-03-23

**Authors:** Federico Bruno, Vincenza Granata, Flavia Cobianchi Bellisari, Ferruccio Sgalambro, Emanuele Tommasino, Pierpaolo Palumbo, Francesco Arrigoni, Diletta Cozzi, Francesca Grassi, Maria Chiara Brunese, Silvia Pradella, Maria Luisa Mangoni di S. Stefano, Carmen Cutolo, Ernesto Di Cesare, Alessandra Splendiani, Andrea Giovagnoni, Vittorio Miele, Roberto Grassi, Carlo Masciocchi, Antonio Barile

**Affiliations:** 1 Department of Biotechnological and Applied Clinical Sciences, University of L’Aquila, 67100 L’Aquila, Italy; flavia.cobianchi@gmail.com (F.C.B.); ferrucciosgalambro@gmail.com (F.S.); emanuele.tommasino@gmail.com (E.T.); alessandra.splendiani@univaq.it (A.S.); carlo.masciocchi@univaq.it (C.M.); antonio.barile@univaq.it (A.B.); 2 Italian Society of Medical and Interventional Radiology (SIRM), SIRM Foundation, 20122 Milan, Italy; palumbopierpaolo89@gmail.com (P.P.); dilettacozzi@gmail.com (D.C.); pradella3@gmail.com (S.P.); 3 Division of Radiology, Istituto Nazionale Tumori IRCCS Fondazione Pascale—IRCCS di Napoli, 80131 Naples, Italy; v.granata@istitutotumori.na.it; 4 Abruzzo Health Unit 1, Department of Diagnostic Imaging, Area of Cardiovascular and Interventional Imaging, 67100 L’Aquila, Italy; 5 Emergency and Interventional Radiology, San Salvatore Hospital, 67100 L’Aquila, Italy; arrigoni.francesco@gmail.com; 6 Division of Radiodiagnostic, Azienda Ospedaliero-Universitaria Careggi, 50134 Firenze, Italy; vmiele@sirm.org; 7 Department of Precision Medicine, University of Campania “L. Vanvitelli”, 81100 Naples, Italy; francescagrassi1996@gmail.com (F.G.); roberto.grassi2013@gmail.com (R.G.); 8 Department of Medicine and Health Sciences “V. Tiberio”, University of Molise, 86100 Campobasso, Italy; mariachiarabrunese@libero.it; 9 Dipartimento di Laboratorio di Analisi e dei Servizi, Azienda Sanitaria Locale Napoli 3 Sud, 80059 Napoli, Italy; dip.diagnostica.immagini@aslnapoli4.it; 10 Department of Emergency and General Surgery, University of Salerno, 84100 Salerno, Italy; verlag@hogrefe.de; 11 Department of Life, Health and Enviromental Sciences, University of L’Aquila, 67100 L’Aquila, Italy; ernesto.dicesare@univaq.it; 12 Departement of Radiological Sciences, Ospedali Riuniti Ancona, Università Politecnica Delle Marche, University Hospital, Via Tronto 10, 60126 Ancona, Italy; a.giovagnoni@univpm.it

**Keywords:** nanomedicine, nanoparticles, magnetic resonance imaging, dynamic contrast enhancement, diffusion weighted imaging, contrast media

## Abstract

**Simple Summary:**

Magnetic Resonance Imaging (MRI) is a consolidated imaging tool for the multiparametric assessment of tissues in various pathologies from degenerative and inflammatory diseases to cancer. In recent years, the continuous technological evolution of the equipment has led to the development of sequences that provide not only anatomical but also functional and metabolic information. In addition, there is a growing and emerging field of research in clinical applications using MRI to exploit the diagnostic and therapeutic capabilities of nanocompounds. This review illustrates the application of the most advanced magnetic resonance techniques in the field of nanomedicine.

**Abstract:**

In the last decades, nanotechnology has been used in a wide range of biomedical applications, both diagnostic and therapeutic. In this scenario, imaging techniques represent a fundamental tool to obtain information about the properties of nanoconstructs and their interactions with the biological environment in preclinical and clinical settings. This paper reviews the state of the art of the application of magnetic resonance imaging in the field of nanomedicine, as well as the use of nanoparticles as diagnostic and therapeutic tools, especially in cancer, including the characteristics that hinder the use of nanoparticles in clinical practice.

## 1. Introduction

Nanomedicine is the application of the principles and methods of nanoscience and nanotechnology to medicine, with the aim of developing more sensitive and faster medical methods and of understanding the processes and mechanisms of life activities at the micro or nano level [1,2,3,4,5,6]. Today, nanotechnology is involved in a wide range of biomedical applications as well as vaccine and drug delivery, imaging, nanosensors, nano-assisted therapies, and engineering [7,8,9,10,11,12,13]. Nanotechnology has advanced rapidly in recent years, bringing significant benefits by treating diseases with greater accuracy and efficacy and by enabling new methods for tumor theranostics [14,15,16]. Nanoparticles integrating imaging, targeting, and therapeutic agents in a single instrument are more useful than separate therapeutic or diagnostic agents [17,18]. In addition, these vehicles are easily transported through leaky blood vessels and the lymphatic system into tumor tissue, either passively (EPR effect) or in conjunction with active targeting agents. The EPR effect improves the access and accumulation of these agents in cancer tissues and increases their efficacy [19,20]. Although oncology is the main area of nanotechnology research, there is growing awareness of the potential use of nanotechnology in non-oncology areas [21].

There has been an important development in the discovery of nanomaterials since they have been introduced as tools for drug delivery. A new, revolutionary way of using nanoparticles is magnetic resonance imaging (MRI) [12,22,23]. The basis of MRI is the interaction between radiofrequency pulses and magnetic moments of hydrogen atoms [24,25]. To achieve this interaction, a high, static magnetic field is required [26,27]. MRI is commonly used in clinical settings because it provides morphologic data with high spatial resolution and enables the assessment of functional parameters such as perfusion, water diffusion, and many others [18,28,29,30,31,32,33]. Recently, the improvement of high-sensitivity receiving coils, especially cryogenically cooled coils, and the use of higher magnetic field strengths have enabled microimaging with spatial resolution as low as 20–50 μm in small animals [34]. The purpose of this article is not only to summarize the status of the application of MRI in the field of nanomedicine but also to analyze the characteristics that hinder the use of nanoparticles in the clinical setting.

### 1.1. Nanoparticles

Inorganic nanomaterials, such as silica, iron oxide, and gold, are increasingly used in nanotechnology, especially as diagnostic vehicles, drug delivery devices, and hyperthermia tools [35,36,37,38]. Based on their composition, they can be divided into organic (lipids, polymers, liposomes, polymeric micelles, dendrimers, engineered peptides, and nucleic acids) and inorganic agents (carbon nanoparticles, metals, and metal oxide nanoparticles). It is believed that linking agents into a single nano-platform can improve the physical properties of the individual agents [39,40]. These combined agents maintain the physical properties of each component agent when linked with the bifunctional agents. Nowadays, nanomaterials are at the forefront of inorganic nano-theranostics, especially in imaging as contrast agents in MRI, in therapy as drug carriers, and as hyperthermia agents [15,41,42,43,44,45].

Nanoparticles can be used as drug carriers to improve the pharmacokinetics of drug delivery and reduce systemic toxicity. Thanks to the application of specific imaging techniques that exploit the physical, chemical, and optical properties of nanomaterials, it is possible to obtain imaging information about drug delivery to the target tissue noninvasively or to achieve thermal and photocontrolled drug release [46,47].

The ability to relate to the material at the nanoscale through external stimuli is of great importance.

**Figure 1:**
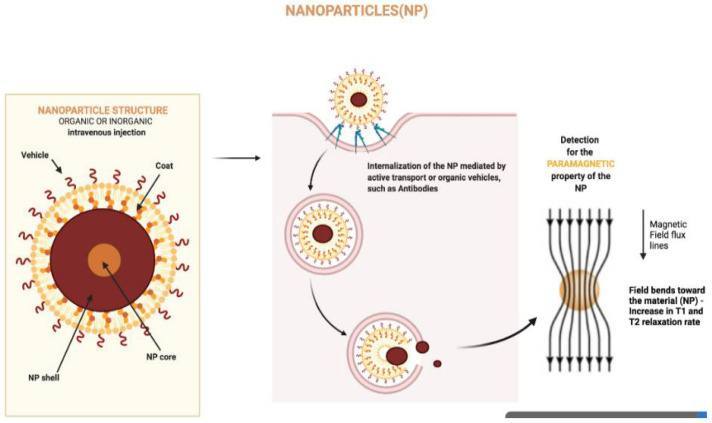
Nanoparticle structure and interaction with magnetic fields. The figure was created with BioRender.com.

An externally generated magnetic field can control the flow and release of magnetic nanomaterials, making them functional diagnostic and therapeutic tools. Various magnetic nanoparticles have been used, but a particular focus has been on small nanoparticles composed of iron oxide, called superparamagnetic iron oxide nanoparticles [35,48]. Superparamagnetic nanomaterials have excellent magnetic properties because they combine the high magnetization of bulk magnetite with the paramagnetic nature of Fe ions. Outside of a magnetic field, these nanomaterials have no magnetic moments [49]. In contrast, if a magnetic field is applied, the magnetic moments align with the field and behave like a paramagnet, but with a higher magnetic susceptibility [50,51] (Figure 1).

Plasmonic nanoparticles are also used in nanotheranostics [52,53]. Plasmons refer to collective oscillations of electrons surrounding the material. At optical frequencies near optical resonance, this local electron density oscillates with the incident electromagnetic excitation. This phenomenon can be used in various fields, such as diagnostics and drug delivery. In addition, they are useful hyperthermia tools for photothermal treatment [54]. Carriers such as gold (plasmonic material) can release energy in the form of heat, which then diffuses into the environment when excited by light near or at their optical resonance frequency [55,56].

### 1.2. Magnetic Resonance Imaging

Imaging is an important tool for tumor assessment in both preclinical and clinical settings [57,58,59,60,61,62,63]. It provides data on the tumor’s size, location, and relationship to adjacent tissues. In addition, imaging can provide data on the biological characteristics of cancers [64], making it possible to assess tumor biology directly in vivo [65,66,67,68]. Because modern cancer therapies essentially target the recognized biological features of the cancer, imaging provides noninvasive data that can be used both to improve drugs and to assist in therapeutic management [69].

MRI has now become a fundamental tool in oncology research and in the clinical management of cancer patients. Among imaging modalities, MRI provides unique and multiparametric access to anatomic, physiologic, biochemical, and molecular details of tumors with excellent spatial and temporal resolution [70,71,72,73,74,75,76,77,78,79].

Conventional (or standard) MRI imaging is based on longitudinal relaxation (T1), transverse relaxation (T2), and proton density (PD) sequences [64]. The technique for image intensity and contrast in standard MRI is based on the relaxation properties of water protons and total water content in tissues [80,81]. Free water shows a dark signal in T1-weighted (-W) images and a bright signal in T2-W images [64,70]. The PD image is related to water content because the image is acquired with minimal T1-W and T2-W to eliminate signal loss due to T1 and T2 relaxations [31,82].

## 2. Functional Magnetic Resonance Imaging

It is well known that a visual inspection of morphologic factors provides only partial data on tissue characteristics [73,77]. Advances in MR technology link morphologic data with functional data about the biological microenvironment of the tissue [83,84]. Functional MR data provide quantifiable information about underlying tissue characteristics [44,64]. The combination of objective biomarkers with morphologic data makes functional MRI a powerful tool that provides comprehensive information about lesion heterogeneity and therapy-induced changes in heterogeneity [28,85,86]. Functional MRI has additional potential in the early stages of treatment efficacy evaluation and should be useful in drug development [86]. Some functional analyses are already part of clinical practice: diffusion-weighted MRI (DW-MRI) and perfusion imaging (DCE-MRI) [13,45,68]. Other technologies, such as metabolic imaging with MRI, are still in the experimental phase.

### 2.1. Diffusion-Weighted Imaging MRI

**Figure 2:**
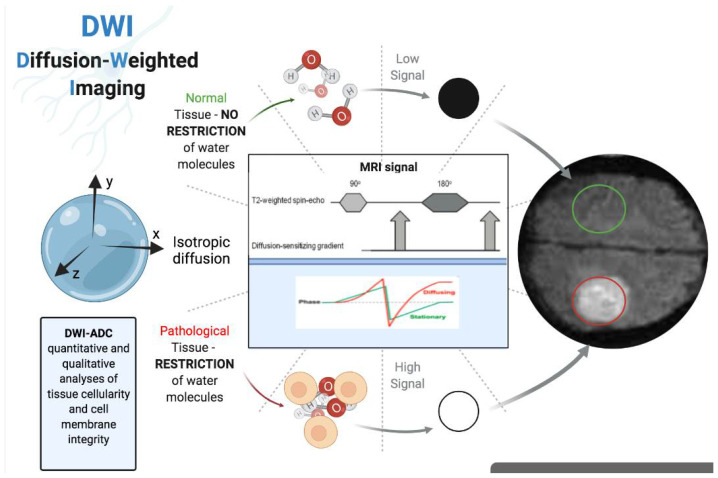
Physical principles of diffusion-weighted imaging (DWI). The figure was created with BioRender.com.

DWI provides quantitative information about tissue microstructure based on differences in water proton mobility and cell density assessment [87,88]. The random movement of water molecules is related to the extent of the cellularity of the tissue as well as to intact cell membranes, and the apparent diffusion coefficient (ADC) is a parameter for quantitative assessment [89,90] (Figure 2). Intravoxel incoherent motion (IVIM) assessment, a bi-exponential model for evaluating a DWI signal, enables the acquisition of the pure tissue coefficient (Dt), the pseudodiffusion coefficient (Dp), and the perfusion fraction (pf) [91]. Conventional DWI assessment is based on the theory that water diffusion in voxels obeys a Gaussian law [92]. In tissues, diffusion is affected by the presence of molecular obstacles and deviates from Gaussian law. To describe this deviation from the Gaussian distribution, a mathematical model known as diffusion kurtosis imaging (DKI) was proposed by Jensen et al. in 2005. The quantitative parameters extracted by DKI are the mean of the mean kurtosis coefficient (MK), which reflects the deviation of tissue diffusion from a Gaussian model, and the mean of the diffusion coefficient (MD), which assesses the correction for non-Gaussian bias [93,94].

### 2.2. Dynamic Contrast-Enhanced-MRI

DCE-MRI guarantees the possibility of obtaining information about tissue perfusion and microvascular structure [57]. This is achieved by analyzing the SI changes in a tissue after the introduction of a contrast agent [95,96,97]. DCE-MRI can assess tissue perfusion and the microvascular status of tissues. It has also been used to visualize changes in tumor perfusion caused by therapies [98,99,100]. The clinical significance of DCE-MRI in predicting and evaluating the response to therapy and the optimal methodology remains to be determined [96,101]. Perfusion analysis can be performed quantitatively, semiquantitatively, or qualitatively [96,97].

Quantitative assessment involves the evaluation of the pharmacokinetic parameters of a contrast agent [102]. The main parameter evaluated is Ktrans, which represents the leakage of the contrast agent between the blood plasma and the extravascular extracellular space and reflects the flow and permeability [99,103]. Because Ktrans is associated with many variations and many different methods are reported, quantitative DCE-MRI is challenging to reproduce in a standardized manner because it is highly variable.

Qualitative DCE assessment involves analyzing a kinetic curve by a simple visual assessment, as confirmed by Fusco et al. [104]. One of the limitations of this approach is the ROI placement, which depends on the user [54].

In semiquantitative analysis, the shape of the time-intensity curve (TIC) is de-scripted, which provides information about wash-in, wash-out, and peak enhancement [102]. Therefore, the semiquantitative approach is the most reliable method compared with quantitative or qualitative methods because several critical points are skipped [94].

### 2.3. BOLD-MRI

Hypoxia is a critical point in aggressive cancer biology and resistance to traditional or targeted treatments. There are several factors that cause low oxygen tension, such as the rapid growth of the tumor and the low microvascular density, which leads to a change in tissue morphology and increases the intracellular tension and stiffness of the tissue.

In addition, the tumor is surrounded by an extensive desmoplastic stroma, which contributes to disruption of the structure of tumor vessels and mechanical stress on endothelial cells and thus tumor vessels [105].

Blood oxygenation-dependent imaging (BOLD) is a technique used to produce functional MRI (fMRI) images that are the result of changes in regional blood concentrations of oxyhemoglobin and deoxyhemoglobin, and are a measure of oxygenation [106].

The physical basis of the techniques is based on the iron ions of deoxyhemoglobin, which contain unpaired electrons and have a paramagnetic property that shortens the transverse relaxation time of protons in close proximity [107]. This is reflected in the tissue T2* value, which correlates negatively with deoxyhemoglobin concentration and thus arterial blood PO2 [108].

### 2.4. MR Spectroscopy

MR Spectroscopy (MRS) is an instrument that provides metabolic information through electromagnetic signals in the radiofrequency range generated by atomic nuclei in molecules [109]. Since the surrounding structures determine the electrical environment, the subsequent resonant frequencies affect the chemical groups and molecules present. MRS provides concentrations of some specific metabolites. However, there are still some open questions to be addressed by this tool [110].

First, it is necessary to obtain a homogeneous magnetic field to resolve the fact that the resonance frequencies of the different metabolites are very close to each other. Second, it is difficult to obtain reliable data of good quality from some tissues, e.g., bone-air interfaces.

Finally, metabolites are present in tissues at minimal concentrations compared with water, so their signals are very weak and a certain minimum concentration of metabolites is required [110].

**Figure 3:**
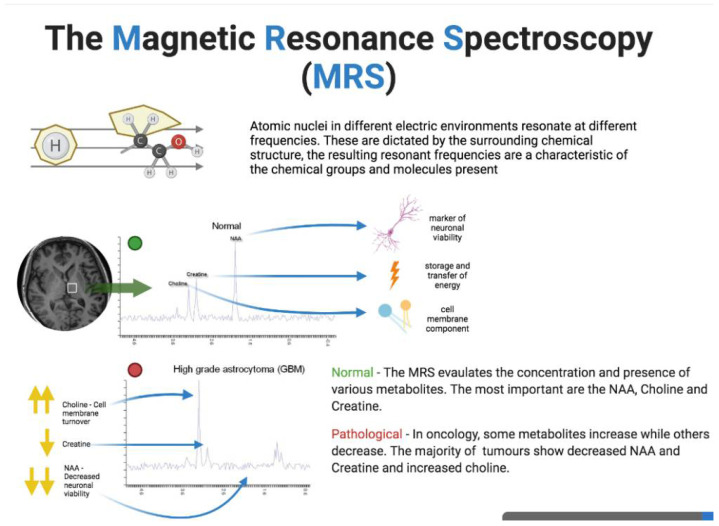
Physical principles of magnetic resonance spectroscopy (MRS). The figure was created with BioRender.com.

Proton MRS (1H MRS) is the most commonly used method for analyzing cancer metabolism. In addition to 1H MRS, other nuclei, such as 19F, 13C, 31P are currently used to evaluate metabolic changes and enzymatic activities in cancer tissues [111] (Figure 3).

## 3. Radiomics

Radiomics is an emerging field in radiology for cancer evaluation [112]. It is well known that radiomic data can be associated with the histologic grade, TNM, response to treatment, and prognosis. The approach of radiomics analysis is based on target segmentation, feature extraction, feature selection, and model building [66,113,114]. Target segmentation is a manual approach and is the major criticism of this process, because it is time-consuming, and because it correlates with variability in target delineation, leading to the problem of reproducibility in feature determination [115,116,117,118].

The data extracted from radiomicroscopy, when combined with other clinical data and correlated with outcome, can create accurate, robust, and evidence-based clinical decision support systems (CDSS) [62,119]. The rationale for radiomics is that quantitative variables based on individual voxels are more sensitive to various clinical endpoints than the qualitative radiologic, histopathologic, and clinical data routinely used in clinical practice [120,121,122,123,124]. An extension of radiomics is radiogenomics, which aims to correlate imaging data with some known genetic predictors of response to therapy and metastatic spread, with potential prognostic utility [92,125]. In this way, radiogenomics could provide the highest level of personalized risk assessment ever developed, making it possible to further advance precision medicine, improve patient selection for various tumor treatments, predict response to therapy and potential resistance, and assess which patients might benefit from adjuvant therapy.

## 4. Nanoparticles and MRI Contrast Agents: Physical Principles and Clinical Setting

MR contrast agents induce static field distortions, affecting the relaxation time of the nuclei. Gadolinium (Gd3+) has a large magnetic dipole moment and seven unpaired electrons; it thus has excellent characteristics to accelerate T1 relaxation of water molecules that are near the Gd metal ion [126].

A local reduction in T1 relaxation (positive contrast) or T2 relaxation (negative contrast) causes an improved image contrast [127]. Since T1 and T2 times are linked to the concentration, the relaxivities are correlated to iron concentration (r1 and r2). The longitudinal relaxivity r1 is influenced by the molecular tumbling time, proton residence lifetime, and coordinating number. In contrast, the transverse relaxivity r2 is proportionate to the square of the magnetic nanoparticle radius and the magnetic saturation [128]. Since the T1 relaxation is correlated to inner-sphere procedures (chemical energy exchange), superparamagnetic nanoparticles are more efficient in T2 uses. In magnetic core–nonmagnetic shell particles, the magnetic field experienced by the water protons (T2) and the degree of chemical exchange (T1) decrease with increased shell thickness.

Additionally, when nanoparticles are assembled, inferior r1 contrast related to lessened surface area and increased r2 contrast related to the magnetic moments’ coupling is supposed. A relaxivity ratio (r2/r1) is frequently employed as an additional evaluation of contrast medium effectiveness. An optimal T2 contrast agent should have a high r2/r1 ratio and a high r2 value. When the ratio is less than 5, the contrast should be chosen as a T1 contrasting; if the ratio is greater than 5, as a T2 contrasting [129].

Nanoparticles have the property of shortening local water T1 or T2, as the electronic magnetic moments of nanomaterial atomic components interact with the nuclear moments of surrounding water molecules [130].

There are several aspects playing a role in the final intensity of the interaction as well as in the MRI signal. One crucial aspect is the strength and the site of the nanomaterial’s magnetic moments and the consequent relation between the crystal core and water components. Furthermore, even if the general rule is that paramagnetic agents shorten both T1 and T2, they can have different effects on relaxation times. In fact, T2 relaxation time is mainly influenced by nanoparticles with strong coupling between atomic components, while T1 relaxation time is affected by paramagnetic atoms or chelates in normal concentrations [94,131].

In this scenario, nanoparticles emerged as potential contrast agents. Larger superparamagnetic iron oxide nanoparticles (SPIO) have the ability to shorten both T2 and T2* relaxation; SPIOS are also taken up by the reticuloendothelial system and are thus suitable for detecting liver lesions [77,132]. They act as negative contrast agents, as they are captured by Kupffer cells, but not by cancer cells [133].

Today, SPIO nanoparticles represent a new field of inquiry, being involved in several stages of scientific development [134].

In order to enhance the signal in magnetic resonance, several methods to hyperpolarize nuclei have been developed. This approach has been recently used to optimize the detection of injected MRI contrast media.

The method used to produce novel, polymer-based contrast media consists of coupling hydrophilic components with paramagnetic agents, such as gadolinium or manganese. Recently, chemical exchange saturation transfer (CEST) has been proposed as a novel system for MRI as well as for 19F-MR, enabling highly sensitive imaging [135]. This approach increases agent relaxivity as well as stability in the blood, while extravasation from the endothelium is limited. Regarding size, the hydrophilic polymer agents are usually smaller than nanoparticles but larger than low molecular weight compounds [136]. The strategy used to prepare a polymer-based MRI contrast medium is based on modifications of functional moieties or site-specificity [137].

Antibodies show improvements when compared to other polymers, in a specific coupling with an antigen. The system currently used to obtain antibody-contrast agents is based on the technique for creating antibody-labeled radioactive probes. Among those polymers, dendrimer-based MRI contrast agents have been the most widely studied [138]. Typically, dendrimers are synthesized using two different methods. On one hand, the branches are prepared separately and thereafter conjugated to the main core. On the other hand, the first step consists of the preparation of the main core. Therefore, dendrimer-based agents consist of a vast array of types, resulting from changes of the main core, the coupling agents, or the branch number [139].

The system to synthetize dendrimer agents consists of a polyethylene glycol (PEG) modification. This combination is particularly useful, as it enhances the effects on relaxivity [140].

New methods have been developed to better couple MRI and intravital microscopy in order to obtain a wide spectrum of information. In fact, thanks to its high spatial resolution, MR enables the depiction of the distribution of the micelles, while it is possible to receive information regarding the cellular and subcellular levels using intravital microscopy [141].

A method was developed to prepare compounds where the MR contrast agent is incorporated into micelles in combination with quantum dots (QDs), with their inherent fluorescence.

The weakness of fluorescence in imaging is limited tissue penetration, but the UV-near infrared (NIR) wavelength can overcome this problem thanks to its good penetration properties [142].

Today, magnetic iron oxide nanoparticles (IONP) or superparamagnetic iron oxide nanoparticles (SPIO) are used in some clinical settings as targeted MRI contrast agents through adjustment with targeting ligands that connect to specific tumor biomarkers [143,144]. For example, antibodies, peptides, or antibody parts coupling with ligands that have a major expression in tumor cells (e.g., EGFR, HER2/neu, αvβ3 integrin, uPAR, and prostate-specific membrane antigen), if linked to IONPs, provide targeted collection and retention of the IONPs in tumor tissues, resulting in T2 contrast for the detection of tumors by MRI [145]. Moreover, receptor-mediated endocytosis enhances the intratumoral transport and retention of particles in cancer cells for the sensitive imaging of drug transport and cancer responses to treatment [146].

## 5. Nanomedicine, Treatment and Magnetic Resonance Imaging Assessment

Nanoparticles allow the delivery of therapeutic agents to a target point, and this new manner to address drugs is called a nanoparticle-based drug delivery system (Nano-DDS) [147]. Nevertheless, one limit of these agents is possible accumulation in the liver instead of targeted tissues [148]. To elude this and delay their circulation, the agents are often attached to polyethylene glycol (PEG) polymer chains on the surface through PEGylation to improve accumulation within the target [149]. Tumor tissues can be targeted passively due to the intensification of cancer vasculature permeability and consequent accumulation of PEGylated nanoparticles of approximately 30–150 nm [150]. In active targeting, nanoparticles with antibody, peptide, or protein coatings connect specifically to the surfaces of cancer cells [151].

Nanomaterial-based drug delivery systems represent a revolutionary tool to suit the desired drug also in a remote site. Doxil is the first FDA-approved nanomaterial-based drug delivery system, with excellent anticancer properties in preclinical studies. However, focusing on clinical performance, Doxil reported superior efficacy over conventional therapy only against a restricted type of cancer cells; this is the consequence of the interaction of drug with tumor processes and tumor complex microenvironments. For instance, a nanomaterial delivery system has a high difficulty of diffusion in the interstitial structure of a pancreatic tumor [152,153]. To surmount the physical obstacle of the stroma, nanoparticles targeting uPAR, which is greatly expressed in cancer cells and tumor-associated stromal cells, have been created. The uPAR-targeted ligand, derived from the amino-terminal fragment (ATF) peptide of urokinase plasminogen activator, was conjugated onto amphiphilic polymer-coated IONPs carrying conditional release chemotherapy drug, Gem (ATF-IONP-Gem). uPAR-targeted ATF-IONP-Gem caused significant growth inhibition in pancreatic tumors [154].

The tissue concentration of nanoparticles in a cancer is correlated to its microstructure. Consequently, nanoparticles cannot always be transported into cancer cells in an adequate dose.

Cancers vary in cellularity and ultrastructure. Other aspects to consider in this scenario are treatment and immune response status. The particles should be selected considering the tumor microenvironment. To realize nanoparticles that amplify the therapeutic effects and reduces unexpected results, it is determinant to assess the morphological characteristics of the nanoparticle drug, the exact dose that can achieve the target, and the manner in which the drug can be transported over a useful time. 3D micro-imaging is essential to see the distribution and therapeutic effects of nanoparticles [155].

IONPs can be linked to anticancer drugs, such as doxorubicin, methotrexate, camptothecin, for therapeutic purposes. Quan et al. reported human serum albumin-stabilized IONPs for the delivery of DOX into tumors guided by MRI, documenting a significant increase in the blood half-life of DOX and drug accumulation in tumors [40].

Faraj et al. assessed magnetic single-walled carbon nanotubes as efficient drug delivery nanocarriers in a breast cancer murine model using DWI, showing that ADC was a sensitive imaging biomarker for the assessment of treatment-induced changes [51,156].

Photothermal cancer therapy (PTT) is another novel treatment that exploits the use of near-infrared (NIR)-irradiated light-absorbing nanoparticles to induce tumor ablation through local hyperthermia. Even if this treatment has been shown to be valuable in various preclinical studies [34], the outcome is variable, depending on several biological features. Therefore, the treatment should be tailored to the individual patient and case [157].

Recently, DWI has been used as a tool for monitoring thermal therapies and to provide insight on tissue damage after injury [89]. Zhang et al. found a time- and temperature-dependent dynamic change of the MRI signal intensity (using T2* WI and ADC map) in tumor microenvironments before any morphological changes due to the effective eradication of tumor blood vessels. Based on the distribution of nanoagents, they also showed that PTT caused a heterogeneous thermal injury of the lesion [126,158].

Fu et al. studied PTT using DWI as a tool for therapy monitoring and early prognosis of treatment. DWI was performed at different time points after PTT, and the tumor ADCs were assessed and compared. They demonstrated that photothermal agents, magnetic guidance, and drug–light intervals could affect PTT efficacy. ADC value changes at early time points after PTT (less than 48 h) were well-correlated with tumor growth suppression. The changes were most sensitive to conditions that can extend survival for more than four weeks, in which cases the 48 h ADC values were increased by more than 80% [87].

Ye et al. found that the combined use of MRI and photoacoustic imaging (PAI) techniques helped them to monitor the vascular permeability and temperature status following treatment, promising to help guide PTT in future translational investigation [151].

Feng et al. investigated the effectiveness of a polydisulfide-based biodegradable macromolecular contrast agent, (Gd-DTPA)-cystamine copolymers (GDCC), in assessing the efficacy of indocyanine green-enhanced photothermal cancer therapy using dynamic contrast-enhanced MRI (DCE-MRI) in breast cancer xenografts in mice. The effectiveness was evaluated by DCE-MRI with a GDCC of 40 KDa (GDCC-40) at 4 h and 7 days after the treatment. The uptake of GDCC-40 by the cancer cells was fit to a two-compartment model to obtain tumor vascular parameters, such as fractional plasma volume (fPV), endothelium transfer coefficient (KPS), and permeability surface area product (PS). The research demonstrated that the fPV, KPS, and PS values of the treated lesions were smaller (*p* < 0.05) than those of untreated lesions at 4 h and recovered to pretreatment values (*p* > 0.05) at 7 days after the treatment [115].

Table 1 summarizes the relevant literature reviewed in the field of nanomedicine and MRI.

## 6. Conclusions

The recent advances in scanners and magnetic resonance sequences enable the increasingly ultrastructural study of body tissues and represent a fundamental tool for the diagnosis and staging of tumor lesions. The ability to exploit the magnetic properties of microparticles, hitherto used in nanomedicine, is opening a new scenario in the application of magnetic resonance techniques to theranostics, with enormous diagnostic and therapeutic potential in different types of cancer.

## Figures and Tables

**Table 1:** Studies assessing MRI application in nanomedicine and theranostics.

Author	Year	Technique	Nanoparticle	Target
Jiang et al. [149]	2019	Magnetic resonance imaging (MRI)/fluorescence probes	Gadodiamide into fluorescent silica nanoparticles (NPs)	Prostate-specific membrane antigen (PSMA) receptor-positive PCa cells
Mason et al. [150]	2021	Hand-held magnetic particle detector and a small-bore MPI scanner	Iron oxide	Breast cancer (intraoperative assessment of tumor margins)
Ye at al. [151]	2018	Magnetic resonance temperature imaging (MRTI) and diffusion-weighted MRI (DWI)	Near-infrared (NIR) irradiated light-absorbing nanoparticles	Monitoring the vascular permeability and temperature status following PTT
Lee et al. [154]	2013	MRI	Urokinase plasminogen activator receptor (uPAR)-targeted magnetic iron oxide nanoparticles (IONPs) + gemcitabine (Gem)	With MRI contrast enhancement by IONPs MRI detection in residual tumors following targeted delivery into uPAR-expressing tumor and stromal cells
Lee et al. [155]	2016	Magnetic resonance imaging (MRI) and real-time upconversion luminescence imaging (UCL)	Transcatheter intra-arterial infusion of targeted multimodal Nd3+-doped upconversion nanoparticle (UCNP)+anti-CD44-monoclonal antibody	Discrimination of liver tumors from normal hepatic tissues in rats
Quan et al. [40]	2011	MRI	Human serum albumin (HSA)-coated iron oxide nanoparticle (HINP) formula + doxorubicin	Tumor suppression effect on 4T1 murine breast cancer xenograft model
Ng et al. [156]	2013	DWI	CRLX101 (cyclodextrin-based polymer particle containing the DNA topoisomerase I inhibitor camptothecin)	Temporal changes in ADC specified early CRLX101 treatment response
Fu et al. [87]	2016	Diffusion-weighted magnetic resonance imaging (DW-MRI)	Photothermal therapy (PTT)	DW-MRI can be an accurate prognosis tool for PTT
Zhang et al. [158]	2015	MRI	Nanoparticle-mediated photothermal therapy (PTT) using graphene oxide (GO)	Time- and temperature-dependent dynamic change of the MRI signal intensity in intratumor microenvironment
Feng et al. [115]	2009	Dynamic contrast-enhanced magnetic resonance imaging (DCE-MRI).	(Gd-DTPA)-cystamine copolymers (GDCC)	DCE-MRI with GDCC-40 is effective for assessing tumor early response to dye-enhanced photothermal therapy and detecting tumor relapse after treatment
